# Identification of cow and buffalo milk based on *Beta* carotene and vitamin-*A* concentration using fluorescence spectroscopy

**DOI:** 10.1371/journal.pone.0178055

**Published:** 2017-05-18

**Authors:** Rahat Ullah, Saranjam Khan, Hina Ali, Muhammad Bilal, Muhammad Saleem

**Affiliations:** Agri. & Biophotonics Division, National Institute of Lasers and Optronics (NILOP), Nilore, Islamabad, Pakistan; Islamic Azad University Mashhad Branch, ISLAMIC REPUBLIC OF IRAN

## Abstract

The current study presents the application of fluorescence spectroscopy for the identification of cow and buffalo milk based on β-carotene and vitamin-A which is of prime importance from the nutritional point of view. All samples were collected from healthy animals of different breeds at the time of lactation in the vicinity of Islamabad, Pakistan. Cow and buffalo milk shows differences at fluorescence emission appeared at band position 382 nm, 440 nm, 505 nm and 525 nm both in classical geometry (right angle) setup as well as front face fluorescence setup. In front face fluorescence geometry, synchronous fluorescence emission shows clear differences at 410 nm and 440 nm between the milk samples of both these species. These fluorescence emissions correspond to fats, vitamin-A and β-carotene. Principal Component Analysis (PCA) further highlighted these differences by showing clear separation between the two data sets on the basis of features obtained from their fluorescence emission spectra. These results indicate that classical geometry (fixed excitation wavelength) as well as front face (synchronous fluorescence emission) of cow and buffalo milk nutrients could be used as fingerprint from identification point of view. This same approach can effectively be used for the determination of adulterants in the milk and other dairy products.

## Introduction

Milk and other dairy products remains always a source of interest for all nutritionists because of its significant role in human life. It is generally obtained from cows, buffaloes, goats, camels, ewes etc. The most important nutritious components of milk are proteins, carbohydrate, vitamins and fats [1]. Among all proteins, Beta-lactoglobulin (β-lg) is a major protein that exists at the normal pH of bovine milk which account for approximately 10–15% of total milk proteins and about 60% of whey protein [2, 3]. Apart from other essential elements, milk is a rich source of carotenoids. Carotenoids are synthesized in plants but not in animals. Beta-carotene (β-carotene) is one of the members of naturally occurring carotenoids and is abundantly available in plants (fruits and vegetables). It is established that β-carotene is present in cow milk but absent in buffalo, goat and ewe milk [4, 5]. In fact, these animals metabolized the carotenoids into vitamin-A and then passed on to milk. β-carotene is also known as pro vitamin-A, as human body easily converts them into retinol (vitamin-A).

In animal foods, dairy products and liver are the rich sources of vitamin-A along with vitamin-A-active carotenoids (β-carotenes in plants) that are partially converted to retinol by animals during or after absorption. Vitamin-A belongs to a group of fat-soluble compounds (retinyl esters) [6]. Vitamins play key role in metabolism as part of enzymes/co-enzymes and as antioxidants, preventing undesired oxidative processes in the body. Vitamin-A is generally available as retinol, retinal and retinoic acid. It is an essential element and is involved in critical biological processes such as cell growth and development, reproduction, vision (sight) and immune system function etc. [7–9]. Reliable information about the composition of milk and dairy products are of great importance and needs to be labeled properly, in order to fulfill consumer’s demands as well as satisfaction.

Different techniques are available which determines milk ingredients/composition as well as detect milk adulterants. Generally, these techniques are time consuming, required sample pre-treatment as well as skilled professionals. These techniques include high-performance liquid chromatography (HPLC), gas chromatography (GC), colorimetric method, polymerase chain reaction (PCR), mass spectrometry etc. [10–13]. In contrast, optical spectroscopic techniques have the potential to be used for reliable analysis of biological samples [14–18]. Among these spectroscopic techniques, the inherent sensitivity of fluorescence spectroscopy provides alternative tool in resolving the biological samples on molecular level. In recent years, fluorescence spectroscopic technique is becoming a valuable tool for the assessment different fruits and vegetables based on their contents like carotenes [19–21]. The main advantage of this technique lies in the fact that it requires no or little sample preparation. Furthermore, it is nondestructive and suits for in-line industrial processing once it’s established.

The main objective of this study is to identify cow and buffalo milk based on β-carotene and vitamin-A fluorescence spectra in combination with multivariate technique. To the knowledge of authors, no research has been reported previously. Although, the fluorescence spectra of cow and buffalo milk samples are clearly separable in both classical geometry (fixed excitation) as well as front face geometry (synchronous fluorescence), the differences between the two data sets have been further highlighted by applying Principal Component Analysis (PCA) to the fluorescence spectral data of both gender’s milk.

## Materials and methods

### Sample collection and preparation

Twenty fresh milk samples each from cow and buffalo have been used in this study. All samples were collected from healthy animals of different breeds at the time of lactation in the vicinity of Islamabad, Pakistan. A portion of about 20 ml whole milk samples were collected of each animal after routine lactation with the consent of dairy farmer. Samples were then transported in air tight tubes in cooled environment to the laboratory. All these samples were then stored without any processing at -16°C temperature in freezer till further use.

### Experimental setup

Spectra from entire milk samples have been recorded using a fully automatic spectrofluorometer system FluoroMax^®^-4 (HORIBA scientific, Jobin Yvon, USA). This system is controlled by a software fluoroEssence^™^ operating within the window environment. FluoroMax^®^-4 is capable of recording spectra in three different modes i.e. excitation, emission and synchronous by modulating emission, excitation or both monochromators. A continuous light source (Ozone free xenon arc-lamp, 150W) works as an excitation source. Spectra from all samples have been recorded under the same experimental conditions. The instrument sensitivity of this fluorescence spectroscopic system in steady state mode is 3000:1. A 4 ml cuvette both quartz and plastic was used in classical geometry (right angle) as well as front face setup. For each sample 10 spectra were recorded under the same conditions.

### Fluorescence spectra and data preprocessing

**Fig 1:**
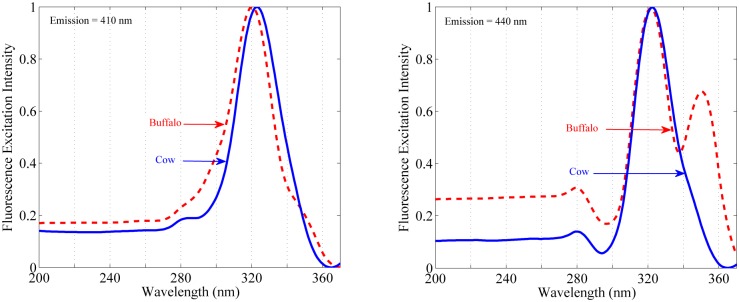
Normalized mean excitation spectra of cow and buffalo milk. *Left*: fluorescence excitation spectra of cow (*solid line*) and buffalo milk (*dash line*) with emission monochromator fixed at 410 nm. *Right*: fluorescence excitation spectra of cow (*solid line*) and buffalo milk (*dash line*) with emission monochromator fixed at 440 nm.

Before making analysis and peak assignment, entire spectral data have been pre-processed and background adjusted for the removal of unwanted contribution to the fluorescence signals. After the background adjustment mean for each individual sample has been calculated. In the next step the mean of each sample has been normalized with the maximum. In Fig 1 is shown the fluorescence excitation spectra of cow and buffalo milk under two fixed emission wavelengths. At emission monochromator fixed at λ_em_ = 410 nm, the excitation spectra of milk of both species gives intense peak at 322 nm and shoulder peaks at 375 nm and 383 nm. Similarly, at fixed emission of λ_em_ = 440 nm, the excitation spectra of cow milk gives intense peak at 322 nm along with their shoulder peaks, whereas, buffalo milk shows two intense peaks at 322 nm and 350 nm.

**Fig 2:**
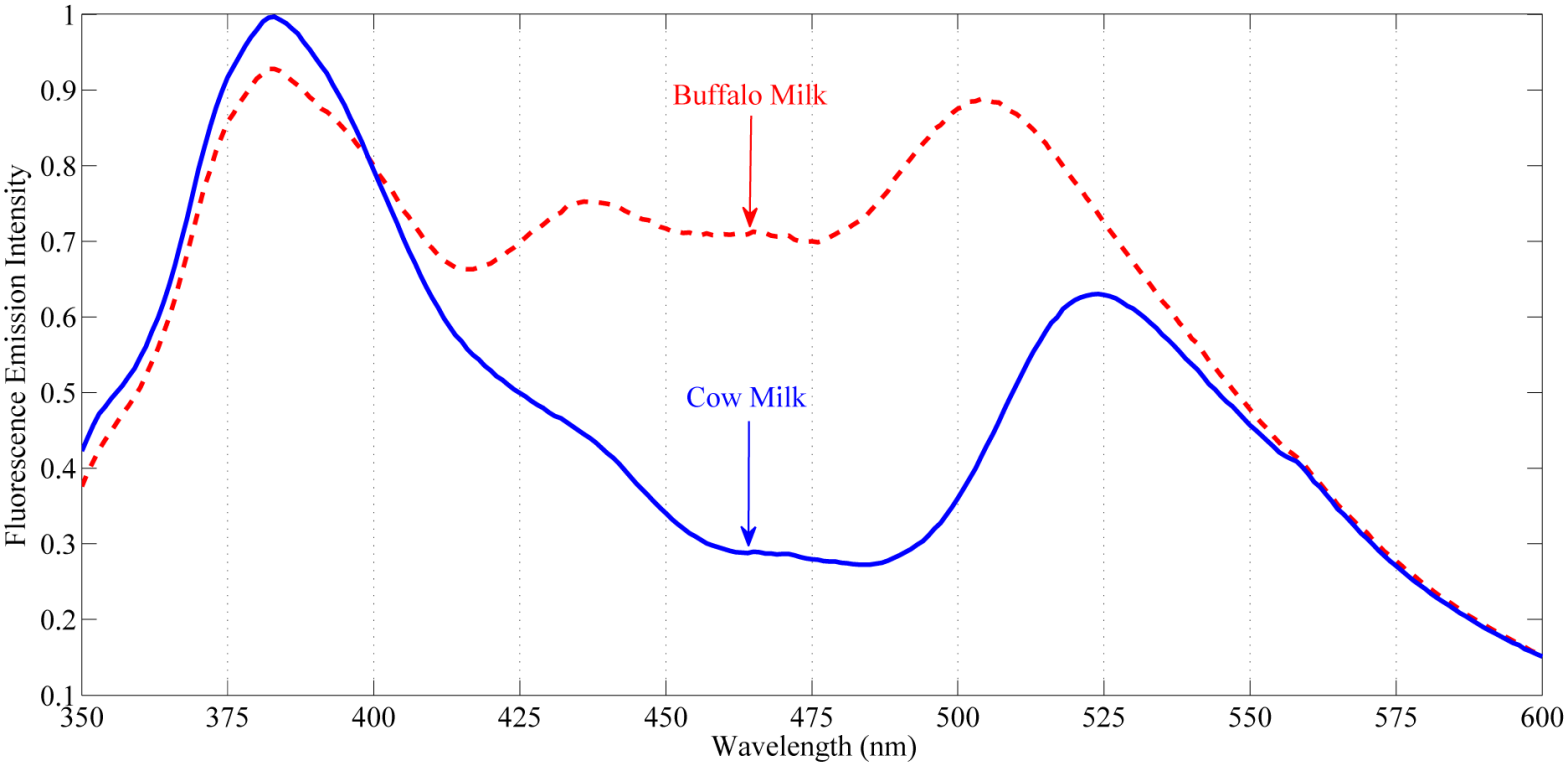
Normalized mean emission spectra of cow and buffalo milk with excitation monochromator fixed at 322 nm. *Top*: fluorescence emission spectra of buffalo (*dash line*). *Bottom*: fluorescence emission spectra of cow (*solid line*).

An excitation wavelength of 322 nm was selected for recoding emission spectra from all milk samples in classical geometry setup. This is because the intense peak at 322 nm appears both in cow and buffalo milk in same conditions. The emission spectral ranging was set from 350–600 nm as this range contains the most essential bio-information of vitamin-A and carotenoids. Fig 2 shows the normalized mean emission spectra for both species with excitation monochromator fixed at 322 nm. For the purpose clear demonstration the emission spectra of cow milk is shown in blue color (solid line) whereas the emission spectra of buffalo is shown in red color (dashed line).

**Fig 3:**
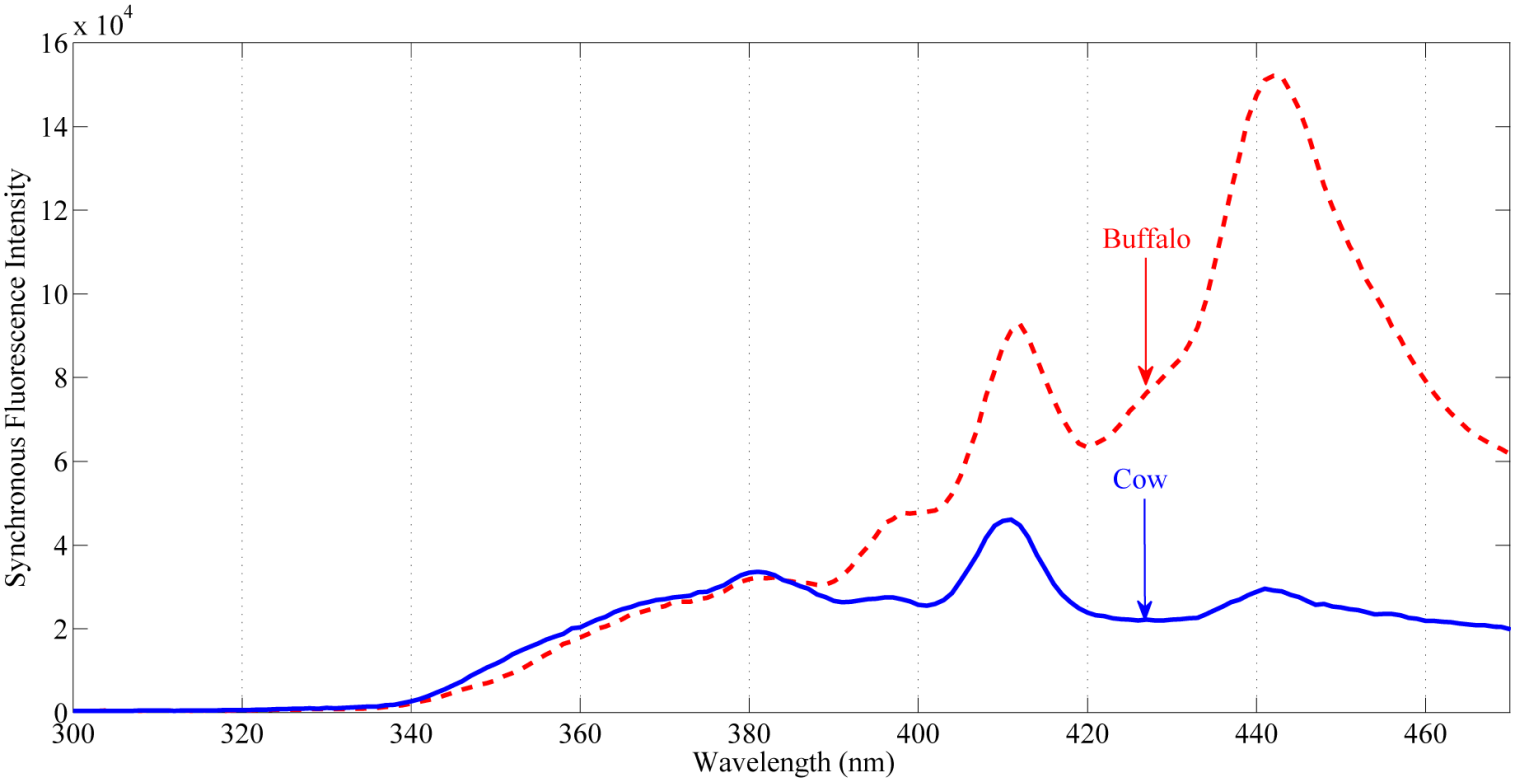
Synchronous fluorescence emission spectra of cow and buffalo milk with offset of 90 nm. *Top*: synchronous emission spectra of buffalo (*dash line*). *Bottom*: synchronous emission spectra of cow (*solid line*).

In synchronous mode, both excitation and emission monochromators are rotated simultaneously by introducing a constant wavelength offset ‘Δλ’ between them. Synchronous fluorescence spectra of cow and buffalo milk (10 spectra from each sample), in the range from 350 nm to 475 nm with an offset of Δλ = 90 nm, have been recorded and shown in Fig 3. In this particular case, the offset was determined empirically by trail. After many trials, an offset of 90 nm provides excellent contrast between cow and buffalo milks. The entrance and exit slits of excitation and emission monochromator’s were set at unity, while the integration interval was set at 0.1 seconds.

### Principal component analysis

Principal component analysis (PCA) is statistical algorithm acting in unsupervised way on the data set. It extracts the meaningful information from the data set and avoids redundant or unwanted spectral information. PCA basically transforms the data into a new co-ordinate system called the principal component (PC) space, whereby each point has new (x,y) values. In the transform domain, the first axis is related to the first principal component (PC1); encompassing maximum variance in the data. Similarly, the second axis which is orthogonal to the first captures the second maximum variance in the data and so on. Additionally, PCA reduces dimensionality in high dimensional data because most of the time, only the first few components (PC’s) explain maximum variation in the data [22, 23].

## Results and discussion

### Identification of milk by classical geometry fluorescence emission

At excitation wavelength of 322 nm, the fluorescence emission spectra of both cow and buffalo milk (Fig 2) show clear differences in position as well as intensities. The fluorescence bands in the emission spectra indicate different naturally occurring fluorophores available in their milk. The emission spectra of both these species show significant variations in the spectral region from 350–600 nm. In this region, the emission peak at around 382 nm arises both in cow and buffalo milk samples having slight variation in intensity. In buffalo milk samples there are additional fluorescence bands at 440 nm and 505 nm which are either completely absent or very low in concentration in cow milk. Similarly, in cow milk an intense emission band at 525 nm can be seen which is absent in buffalo milk.

The distinct fluorescence emission peak appeared at 382 nm both in cow as well as buffalo milk correspond to fats soluble vitamins and other flluorophores like β-carotene [9, 24, 25]. Vitamin-A is fat soluble and due to its conjugated double bond; it is a good fluorophore when excited at 322 nm. Similarly, β-carotene also has emission fluorescence at 382 nm under excitation of 322 nm. Comparison of the emission spectra of both species at this particular band shows higher intensity in cow milk than buffalo. Furthermore, the characteristic fluorescence emission bands at 440 nm and 505 nm indicate the presence of vitamin-A. These results are in accordance with the previous studies which show that vitamin-A gave maximum fluorescence emission at 440 nm when excited at 322 nm [26, 27]. Recently, it has been reported that vitamin-A excitation is interconnected with the physical state of the triglycerides within the fat globules [28, 29]. Comparison of these emission bands (Fig 2) shows that buffalo milk contains vitamin-A, while this compound is either absent or in very low concentration in cow milk.

Moreover, cow milk shows an intense fluorescence band appeared at 525 nm which is absent in buffalo milk. This band corresponds to carotenoids more specifically β-carotene [30]. As reported, buffalo metabolize β-carotene mostly into vitamin-A and then passed on to milk, whereas, in cow milk it is stored in fat globules surrounded by protein cluster. Most probably, the emission at 525 nm by β-carotene is arises due to secondary inner filter effect i.e. transfer of fluorescence between fat residues and β-carotenes may be observed [31]. Since β-carotenes have one of its absorption bands at 380 nm, it is possible that the emitted photons by fat soluble fluorophores at 380 nm are absorbed by these pigments and then re-emits by giving its characteristic emission at 525 nm [32–34]. The re-absorption of emitted photons at 380 nm by β-carotene could be one of the possible reasons for giving emission peak at this particular band in cow milk when compared with buffalo milk samples as seen in (Fig 2).

### Identification of milk by front face synchronous fluorescence emission

Synchronous fluorescence is a complex pattern for the reason that the emission monochromator collects fluorescent emission only when the absorption and emission bands of a biomolecule overlap by the specified wavelength interval ‘Δλ’ [3, 35]. Fig 3 depicts the front face synchronous fluorescence emission intensity results as a function of wavelength interval and excitation wavelength. The synchronous emission spectra of cow and buffalo milk shows intensity variations at bands appeared at 380 nm, 397 nm and 410 nm whereas position variations at 440 nm. In the 400–475 nm range, corresponding to the fluorescence emission of vitamin-A and other fluorophores available in milk, the fluorescence intensities at around 400 nm in cow and buffalo milk are comparable. Similarly, at band position 440 nm very distinctive differences between cow and buffalo milk can be seen.

The synchronous emission at 380 nm (excitation 290 nm) is arises both in cow and buffalo milk are due to tryptophan residues in protein and riboflavin ‘vitamin-B_2_’ [36]. Tryptophan is an important intrinsic fluorescent probe that belongs to the family of amino acids which is used in the biosynthesis of protein. Tryptophan is an essential amino acid which acts as natural mood regulator and helps in fighting against anxiety etc. Slight variation in intensity at band position 380 nm can be seen between buffalo and cow showing higher intensity for the later one (Fig 3). These results show that the concentration of tryptophan residues in protein is in good amount in cow milk when compared to buffalo milk [37, 38].

Furthermore, the intense emission at band positions 410 nm (excitation 320 nm) corresponds to vitamin B_6_ as well as vitamin-A [3, 26]. Both vitamin B_6_ (water soluble fats) and vitamin-A fluoresce at this peak position. Similarly, the synchronous fluorescence emission at band position 440 nm (excitation 350 nm) in cow milk (only shoulder) and in buffalo milk (intense) corresponds to vitamin A [26–28, 39]. Moreover, cow milk contains β-carotenes abundantly and since one of the absorption bands of β-carotenes also lies at round 440 nm, so re-absorption could be the reason for the less intense or only shoulder fluorescence emission at this particular band when compared with buffalo [30, 33]. The intensity variation in fluorescence emission spectra between buffalo and cow (presence and absence of synchronous fluorescence emission at 440 nm) depicts that buffalo milk contains more vitamin-A as compared to cow milk.

**Fig 4:**
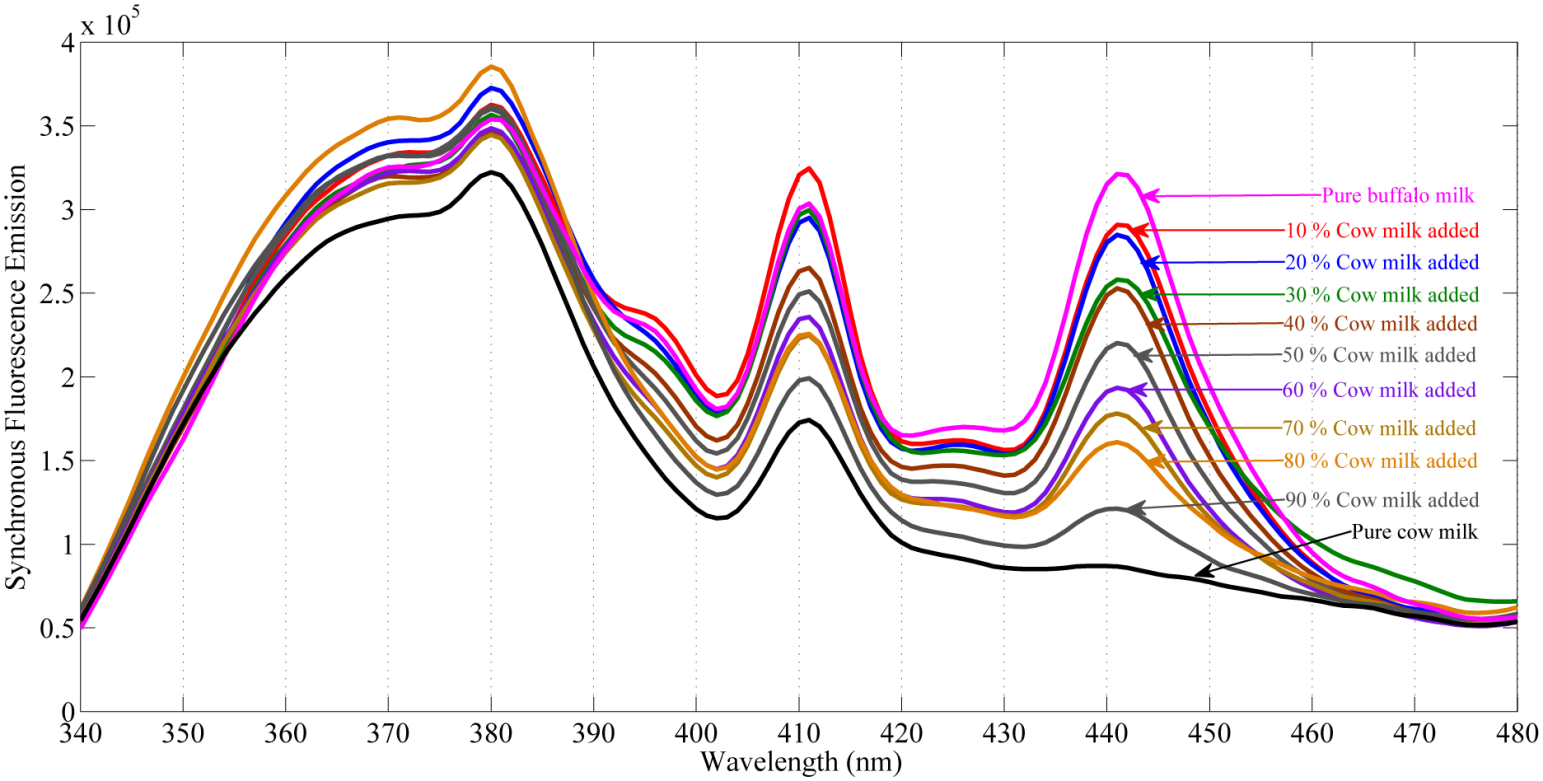
Synchronous fluorescence emission spectra of pure cow and buffalo milk as well as cow milk added to buffalo milk in different concentration with an offset of 90 nm. *Top*: buffalo milk spectra. *Middle*: cow milk added in different concentration to buffalo milk. *Bottom*: cow milk spectra.

Likewise, the synchronous fluorescence emission spectra of pure buffalo and pure cow milk as well as cow milk added to buffalo milk in different concentration is shown in Fig 4. This spectra show intensity variations at band positions of 410 nm and 440 nm. From Fig 4, it is very clear that fluorescence emission at these peak position decreases by increasing the concentration of cow milk in buffalo milk. In both cases i.e. pure milk as well as milk added to each other, the front face synchronous fluorescence emission at band position 440 nm is highly selective and can be used as an indicator to identify cow and buffalo milk.

### Chemometric data classification

**Fig 5:**
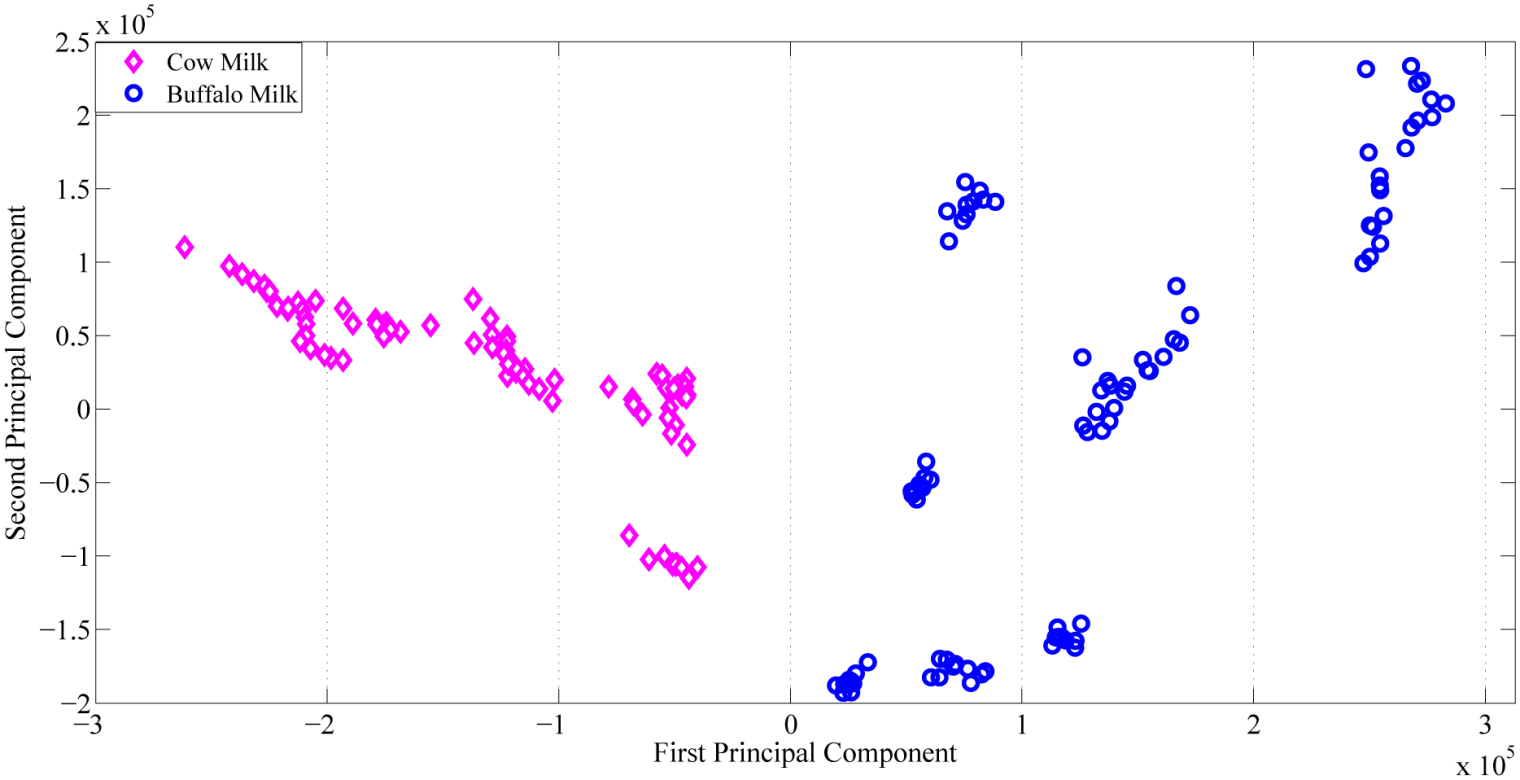
PCA scatter plot of cow and buffalo milk. *Open diamond*: cow milk fluorescence data set. *Open circle*: buffalo milk fluorescence data set.

Fig 5 depicts the PCA scattering plot of cow and buffalo milk. In this figure the first two principal components i.e. PC1 vs. PC2 make a clear difference between the two types of data points (cow and buffalo milk). In the scatter plot of first two PC’s, between the cluster distances is quite good and the data has been separated very well. However, the within cluster divergence can be seen particularly in the buffalo milk. The most obvious reasons for this variation could be infant gender, lactation period, feeds, breed etc. Considering both i.e. between the cluster and within the cluster distances of the data set, it has been found that both are within the desirable limits from identification point of view.

The results reported in this study have not been published and are the first of its kind. From identification point of view, the fluorescence emission spectra of vitamin A and β-carotene of cow and buffalo milk through classical geometry (right angle) as well front face geometrical setup (synchronous fluorescence emission) make it a very good biomarker. Further studies will be extended in order to identify milk of other mammals using this easy and efficient technique. This same approach can effectively be used for the determination of adulterants in the milk and dairy products.

## Conclusion

The current study presents the application of fluorescence spectroscopy for the identification of cow and buffalo milk based on their ingredients which is of prime importance from the nutritional point of view. Cow and buffalo milk shows differences at fluorescence emission appeared at band position 382 nm, 440 nm, 505 nm and 525 nm both in classical geometry (right angle) setup as well as front face fluorescence setup. In front face fluorescence geometry, synchronous fluorescence emission shows clear differences at 410 nm and 440 nm between the milk samples of both these species. These fluorescence emissions correspond to fats, vitamin-A and β-carotene. Principal Component Analysis (PCA) further highlighted these difference by showing clear separation between the two data sets on the basis of features obtained from their fluorescence emission spectra. These results indicate that classical geometry (fixed excitation) as well as front face (synchronous fluorescence emission) of cow and buffalo milk nutrients could be used as fingerprint from identification point of view. This same approach can effectively be used for the determination of adulterants in the milk and other dairy products.
